# Priority setting for the introduction of rotavirus vaccine: what evidence was essential?

**DOI:** 10.1186/s12962-018-0126-7

**Published:** 2018-11-09

**Authors:** Roger I. Glass

**Affiliations:** Fogarty International Center, National Institutes of Health, 31 Center Drive, MSC 2220, Bethesda, MD 20892 USA

**Keywords:** Rotavirus, Vaccines, Vaccine hesitancy, Childhood diarrhea

## Abstract

Rotavirus (RV) diarrhea is the most common cause of severe diarrhea in children worldwide and since 2006, vaccines have been available and recommended by WHO for use in all children. We developed protocols that countries could use to assess the burden of RV disease in their own countries and the cost-effectiveness of a program for vaccine introduction. A decade later and in the setting of extreme tiering of prices so that the poorest countries pay the least for the vaccine, more than 92 countries have introduced this vaccine into their national programs and more than 90 have not. Those countries that introduced determined by protocol that the burden of RV disease was substantial and the cost of vaccine reasonable, especially in low income settings where GAVI subsidizes the vaccines’ purchase. However, elsewhere, WHO's global recommendation has not been enacted leaving a majority of the world’s children still at risk of this severe and sometimes fatal disease. We remain with much to learn about how to encourage countries to make decisions that will improve the health of their own children.

## Background

In considering the value of Multiple Criteria Decision Analysis, I reflected on my own experience creating evidence to encourage countries to introduce rotavirus vaccines into their national program of childhood immunizations and seeing the response from decision makers. Rotavirus is the most common cause of severe diarrhea in children and two new vaccines were licensed in 2006 and subsequently recommended by WHO for the immunization of all children worldwide [1]. As of January 2017, these vaccines have been incorporated into national programs of more than 92 countries, including many in low income settings [2]. What can we learn from the experience of early adopters and late non-adopters of vaccines that might provide insights into how countries have reached their decisions? What information was deemed critical to making these decisions?

To anticipate vaccine introductions, we developed two analytic tools we believed would be critical to allow countries to build their own evidence base to consider vaccine introduction [3]. The first was a protocol for sentinel hospital surveillance for RV so they could assess the burden of RV hospitalizations and deaths at home. The second was a method to estimate the cost-effectiveness (CEA) of a national immunization program based upon local costs and practices [4]. Early on, the two major vaccine manufacturers tiered vaccine prices for middle income countries in Latin America for the PAHO Revolving Fund and through GAVI for low income countries. GAVI further subsidized vaccine purchase for 5 years for those countries having an immunization program capable of absorbing another pediatric vaccine bringing the price down to $0.15–0.30 per dose [5].

As countries began their deliberations, early adopters were self-selected in ways that permit questioning what information was most critical to their making an early decision. Data on the disease burden was useful but not always compelling and fatal cases of RV, as expected, are concentrated in low income countries and rare in high income countries. Consequently, issues of cost-effectiveness, safety, and access to treatment of severe RV diarrhea and any adverse events all played a role in the decision.

In many countries, CEA can be a major driver of the decision to introduce the vaccine. In the US, both direct medical cost and the indirect costs (mostly caregivers’ worktime lost) were factored into the calculation which led to a relatively high valuation for a vaccine program, ~ $200 per child [6]. The UK calculated their CEA without the indirect costs so despite the fact that the incidence of hospital admissions for RV was nearly twice as high as in the US. The UK Department of Health arrived at a much lower value for the vaccine allowing them to negotiate purchase for a price about one-fourth that paid in the US. Australia introduced the vaccine nationwide and did not stop their program even when the complication of intussusception (IS) was linked to the vaccine at a rate substantially greater than in the other countries [7]. Since Australians have good access to care and there has not been a death from IS for years, they decided that the value of the vaccine to reduce hospitalizations for RV diarrhea was enough to justify continuation. European countries have RV in the private market but have been slow to introduce it nationally believing that the disease is generally mild and treatable and that the vaccine remains expensive and not a good buy. Given this ambiguity in the value of the vaccine, other factors have then received greater prominence. In France, a heightened concern over a few cases of intussusception and deaths following vaccination led to its withdrawal from the market. In Spain, the presence of small fragments of DNA from porcine circovirus in one vaccine led to its removal from the market despite no data linking this DNA with an adverse event.

Middle income countries have behaved similarly to the high-income countries balancing the burden of disease locally with the cost of the vaccine relative to other vaccines being purchased or considered for introduction, and not the CEA. In Latin America where many clinical trials were conducted, a tiered price ~ 90% lower than the US price led to early adoption by a majority of the countries, facilitated by a strong recommendation from PAHO which hosts the best regional vaccination program the WHO regions. To facilitate use of the vaccine, the PAHO Revolving Fund purchased the vaccine for a set price making it available to all countries of the region [8]. In the rest of the world, most middle-income countries have chosen not to adopt the vaccine based upon their assessment of the relatively high cost of the vaccine relative to the other vaccines in their portfolios, their perception that the disease is relatively mild and treatable, assessments made often without a CEA. In Thailand, decision makers decided to approve introduction of the vaccine only after its impact could be demonstrated in a local trial that also collected data on the cost of the disease and its care [9]. This decision has delayed introduction for several years and allowed policy makers to track the anticipated decline in the price of vaccine as new suppliers entered the market and have driven down the price. In Malaysia, the evidence of low levels of DNA from porcine circovirus, a contaminant of the trypsin used in vaccine manufacture, raised red flags in this predominantly Muslim country that were unanticipated and delayed introduction.

In the poorest countries, the burden of severe and fatal disease has long made this vaccine a high priority, but the cost of the vaccine initially made it unaffordable. The decisions by WHO to recommend the vaccine for global, use, the companies to severely tier their prices for these markets, and GAVI to purchase vaccine at a tiered price and further subsidize its introduction for 5 years encouraged those countries with the greatest burden of fatal disease and high vaccine coverage to sign on to the program. The rapid expansion of vaccine use in Africa is a direct response to GAVI’s innovative strategy to subsidize vaccine and support its introduction. Whether these countries will continue using the vaccines when the subsidies are discontinued or when the countries graduate to middle income status is a major concern for the future.

In India, the development of two newly licensed vaccines made domestically and purchased for the national immunization program for about $1.00 per dose ($3.00 per child) has led Prime Minister Modi to commit to providing RV vaccine to every infant in India [10]. This decision rested not only on the huge burden of disease, (India has about one-fifth of the RV deaths in the world), the demonstrated safety and efficacy of the vaccines, but also, the fact that the vaccines were made domestically, consistent with a priority for the country to make its own vaccines and be independent of the multi-nationals.

Initially, we believed that assessing the burden of disease would be an essential driver for the early acceptance of vaccines and felt that countries with the best data early on might be the first to adopt the vaccine. Global surveillance for RV surveillance was established first among 10 countries of Asia with GDPs ranging rich to poor [11]. To date, this region has been among the slowest to introduce RV vaccines. Perhaps the absence of clear local champions, the lackluster support from the WHO regional offices, the need for more granular data on the burden of disease, concern over the relatively high price of the vaccine outside of the GAVI countries, have all played a role in the delayed adoption seen throughout the region. It is here that MDCA might have its greatest impact and fill in for the failure of other methods to make a difference.

## Conclusion

Today, a decade after rotavirus vaccines have become universally available, a majority of countries have still not embraced their use in their national programs although the roll out is still a work in progress. Clearly, analytic tools are helpful but ultimately, the price of the vaccine, perception of the severity of the disease, and behavior of programs in neighboring countries, will all play a critical role to accommodate WHO’s global recommendation for universal coverage. Data on the burden of disease and the cost of the vaccine while both are key drivers of the decision, are still not adequate to address the idiosyncrasies of the decision-making process to include RV vaccine in national immunization programs. Further work on analytics such as the MCDA, studies of why countries define themselves as early vs. late adopters, and the value of local vaccine champions or anti-vaccine antagonists could provide additional insights on the path to achieve universal coverage.
